# Supplementary material for: Self-healable printed magnetic field sensors using alternating magnetic fields
Source: Nat Commun. 2022 Nov 3;13:6587. doi: 10.1038/s41467-022-34235-3 (PMC9631606; doi:10.1038/s41467-022-34235-3)
Supplement: Supplementary file 1 — supplementary information [file 41467_2022_34235_MOESM1_ESM.pdf]

Self-healable printed magnetic field sensors using alternating magnetic fields

Authors: Rui Xu[1*], Gilbert Santiago Cañón Bermúdez[1], Oleksandr V. Pylypovskyi[1,2], Oleksii M. Volkov[1], Eduardo Sergio Oliveros Mata[1], Yevhen Zabila[1], Rico Illing[1], Pavlo Makushko[1], Pavel Milkin[3], Leonid Ionov[3], Jürgen Fassbender[1], Denys Makarov[1]*

*Email: r.xu@hzdr.de (RX); d.makarov@hzdr.de (DM)

Affiliations: [1]Helmholtz-Zentrum Dresden-Rossendorf e.V., Institute of Ion Beam Physics and Materials Research, Bautzner Landstrasse 400, 01328 Dresden, Germany; [2]Kyiv Academic University, Kyiv 03142, Ukraine; [3]Bavarian Polymer Institute, University of Bayreuth, Ludwig Thoma Str 36a, 95447 Bayreuth, Germany.

This PDF file includes:

Supplementary Figs. 1 to 33

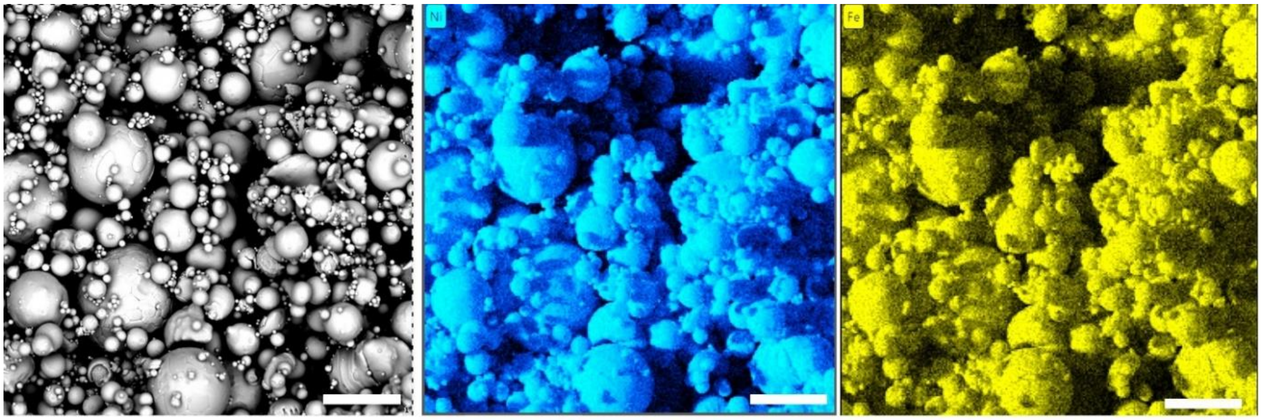

Fig. S1 Energy-dispersive X-ray Spectroscopy (EDX) maps of $Ni_{81}Fe_{19}$ microparticles. Elements of Ni and Fe distribute uniformly over the microparticles. Scale bars: 30 μm.

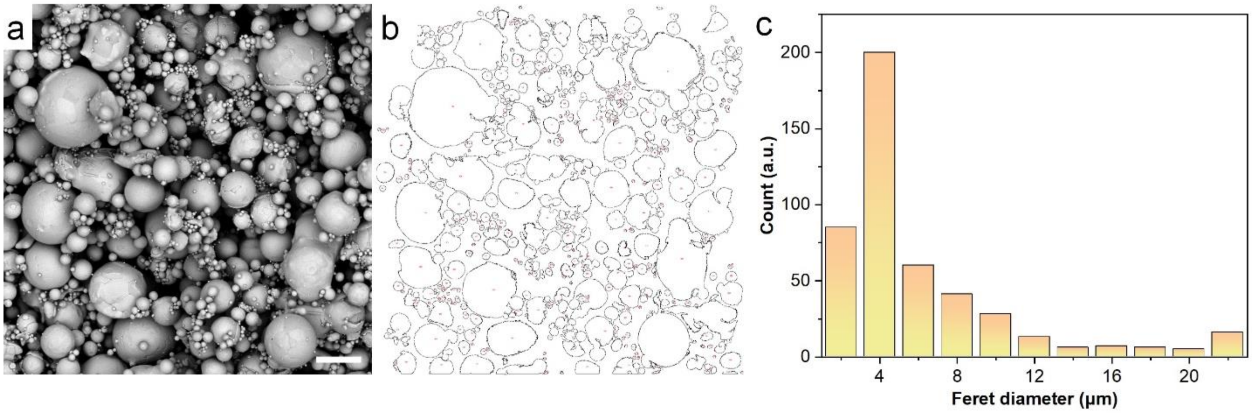

Fig. S2 Statistical analysis of $Ni_{81}Fe_{19}$ microparticles. a) SEM image. b) Outlined profiles of $Ni_{81}Fe_{19}$ microparticles. c) Distribution of microparticle diameters. Scale bar: 20 μm.

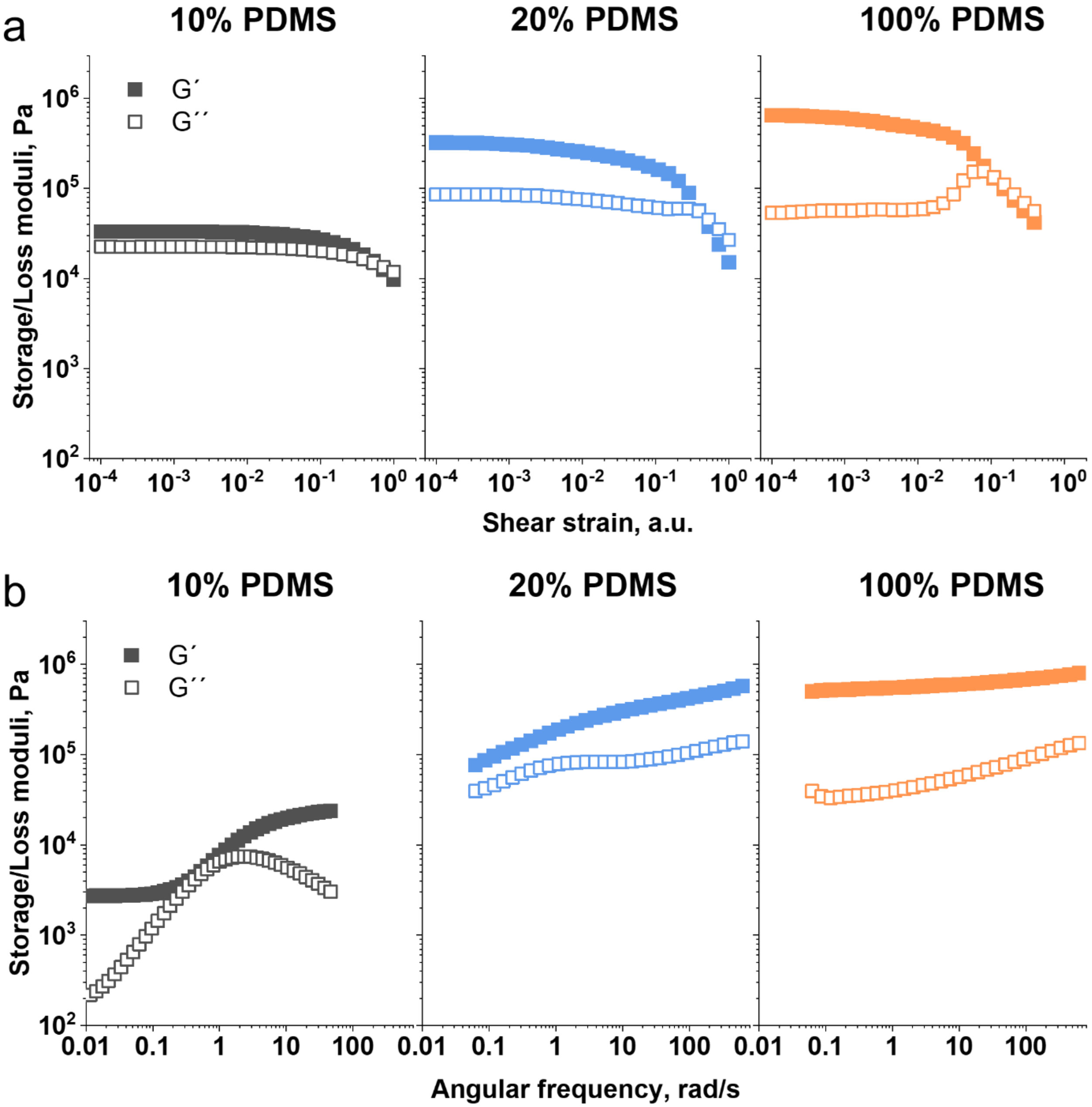

Fig. S3 Dynamic mechanical analysis (DMA) of cured composites. a) Amplitude sweep characterization (at a frequency of 10 rad/s) for the composites with different polymer contents (from left to right): PDMS : PBS = 10 : 90, PDMS : PBS = 20 : 80, pure PDMS. b) Viscoelastic behavior characterization at small deformations.

The results confirm the elastic behavior of all samples, given that the storage moduli are higher than loss moduli over the entire frequency range. The elasticity for the composite made of pure PDMS is due to covalently crosslinked polymer networks. The addition of high amount of PBS introduces the relaxation processes of the composite around 1 rad/s. This behavior is caused by the relaxation of supramolecular network of PBS. The higher amount of PBS, the more pronounced the effect of PBS relaxation. Pure PBS behaves as viscous liquid at long time scale and flows.

Fortunately, even 10% of crosslinked PDMS can provide mechanical stability[2]. Note that, the microparticle volume fractions of the measured composites are only about 12%. With the addition of more microparticles, the mechanical stability can be further enhanced due to the confinement of microparticles matrix[3].

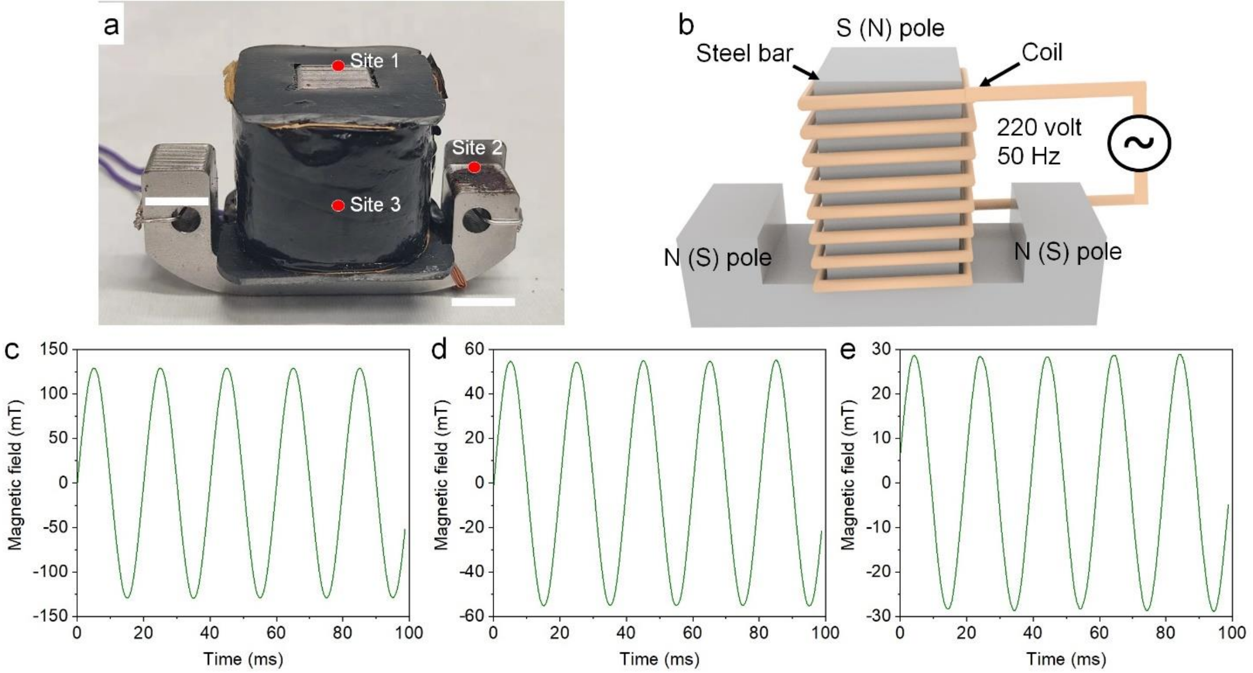

Fig. S4 Characterization of the AMF setup. a) Photograph and b) schematic illustration of solenoid used for generating AMF. c-e) Temporal evolution of the magnetic field measured at different locations, *e.g.*, sites 1 – 3 indicated in a). A commercial Hall-effect sensor was used for calibration.

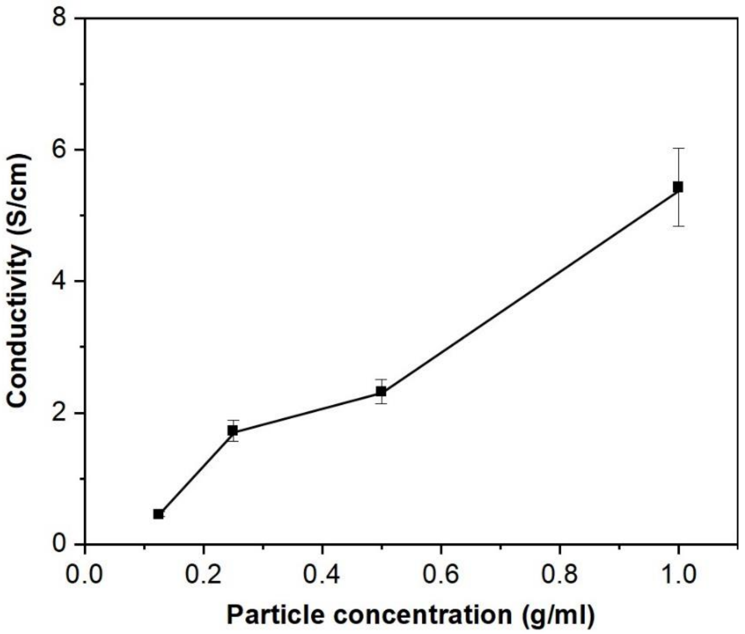

Fig. S5 Electrical conductivity of magnetoresistive composite owning different concentrations of $Ni_{81}Fe_{19}$ microparticles. For assuring the measurement uniformity, magnetoresistive composites were printed on commercial flat flexible cables. Each cable has ten measurement pads and the inter-pad distance are about 0.4 mm. Two-point configuration was used to measure the electrical conductivity. In other words, nine conductivity values were measured for every type of composite. The composite thicknesses were about tens of to hundreds of micrometers.

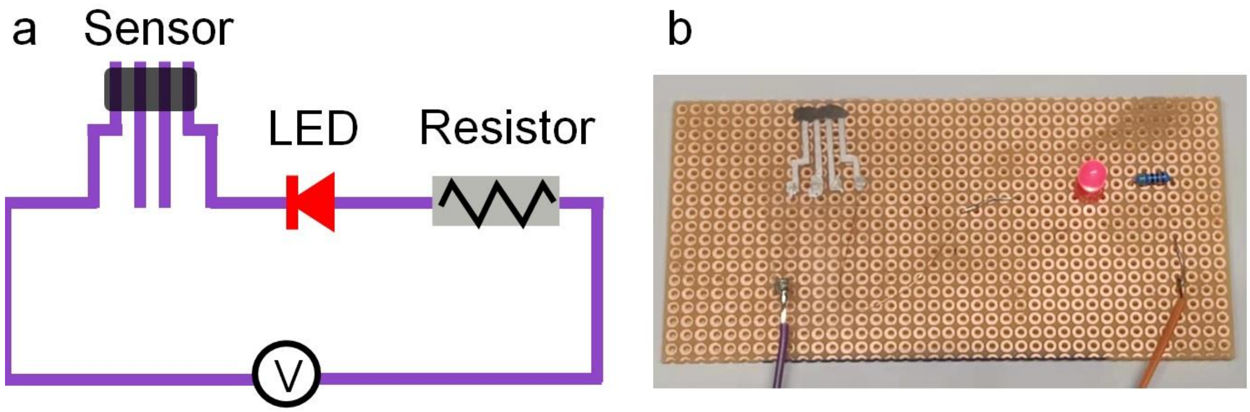

Fig. S6 Electrical circuit consisting of a printed magnetoresistive sensor, a LED, and a resistor. a) Schematic illustration. b) Photograph of all elements on printed circuit board. As of the sensor, the applied composite solution had the 1 g/ml concentration and the conductive pad was made of commercial conductive silver composite.

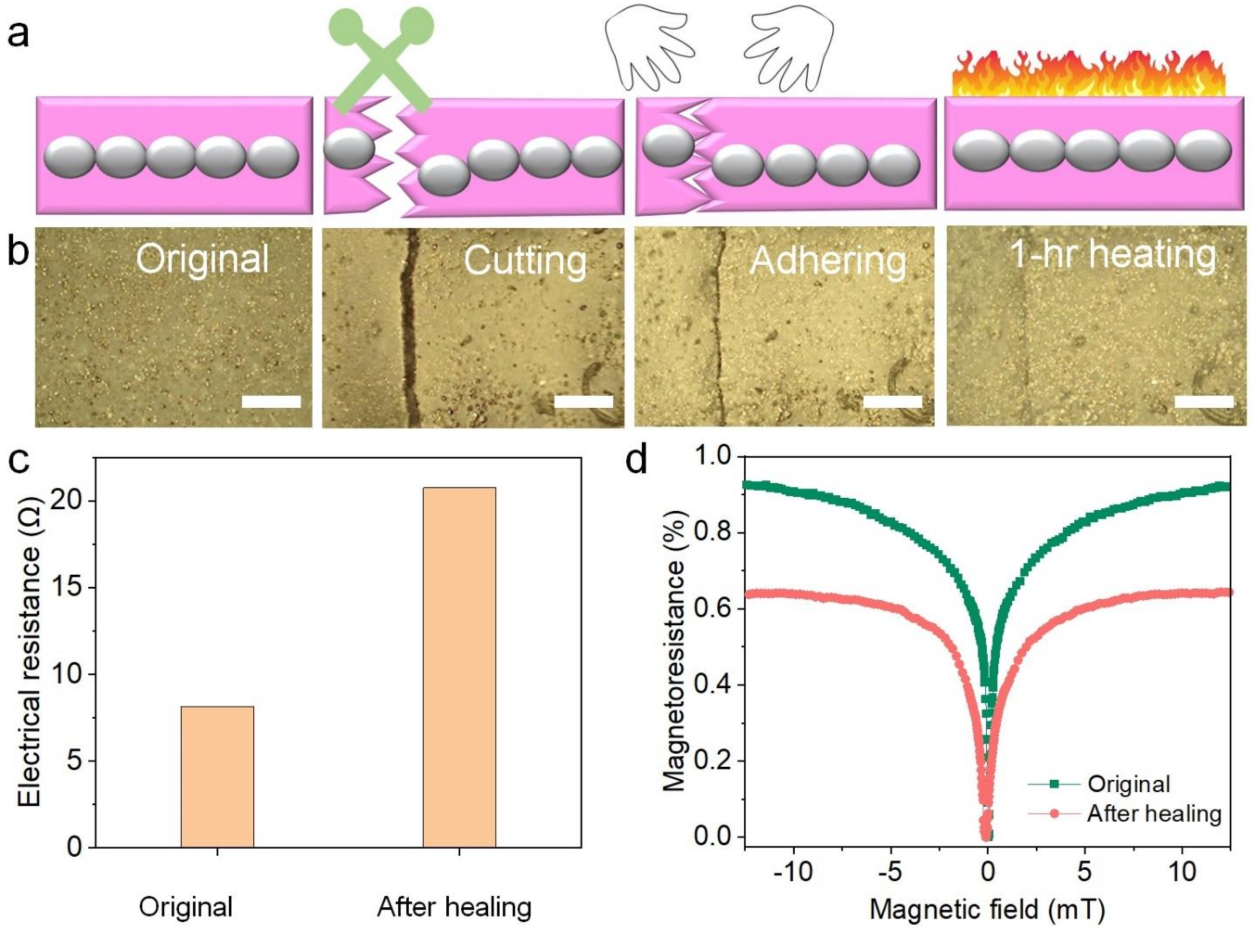

Fig. S7 Self-healing of magnetoresistive sensor by thermal treatment. a) Schematic illustration and b) microscopic photograph of magnetoresistive composite (from left to right): original, cutting by a blade, manual reassembly, and heating for 60 min at 120°C. Scale bars: 200 µm. c) Electrical resistance and d) magnetoresistance of the sensor before and after healing. The magnetoresistive sensor was fabricated on Si wafer by pipetting composite solution with 0.4 g/ml of $Ni_{81}Fe_{19}$ microparticles. Other fabrication procedures were the same with that stated in the Methods section.

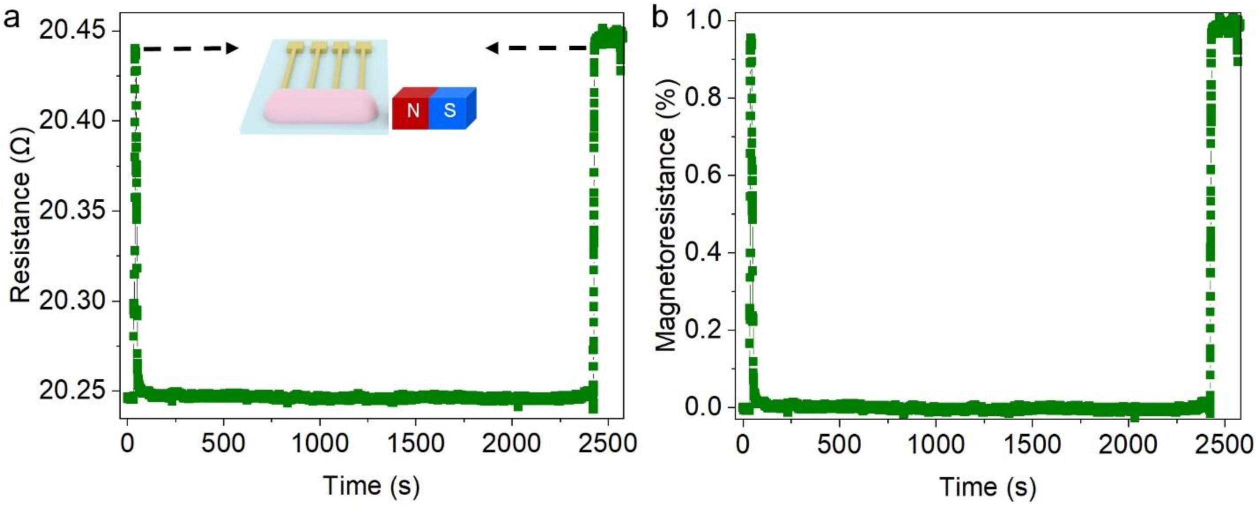

Fig. S8 Operational stability of a magnetoresistive sensor after AMF-mediated self-healing. Continuous measurement of the a) electrical resistance and b) magnetoresistance as a function of time. Inset: Configuration of measurement. The resistance was increased when the sample was exposed to magnetic field. The sensor response to magnetic field was stable after the self-healing process and did not change even after 2500 s of measurement.

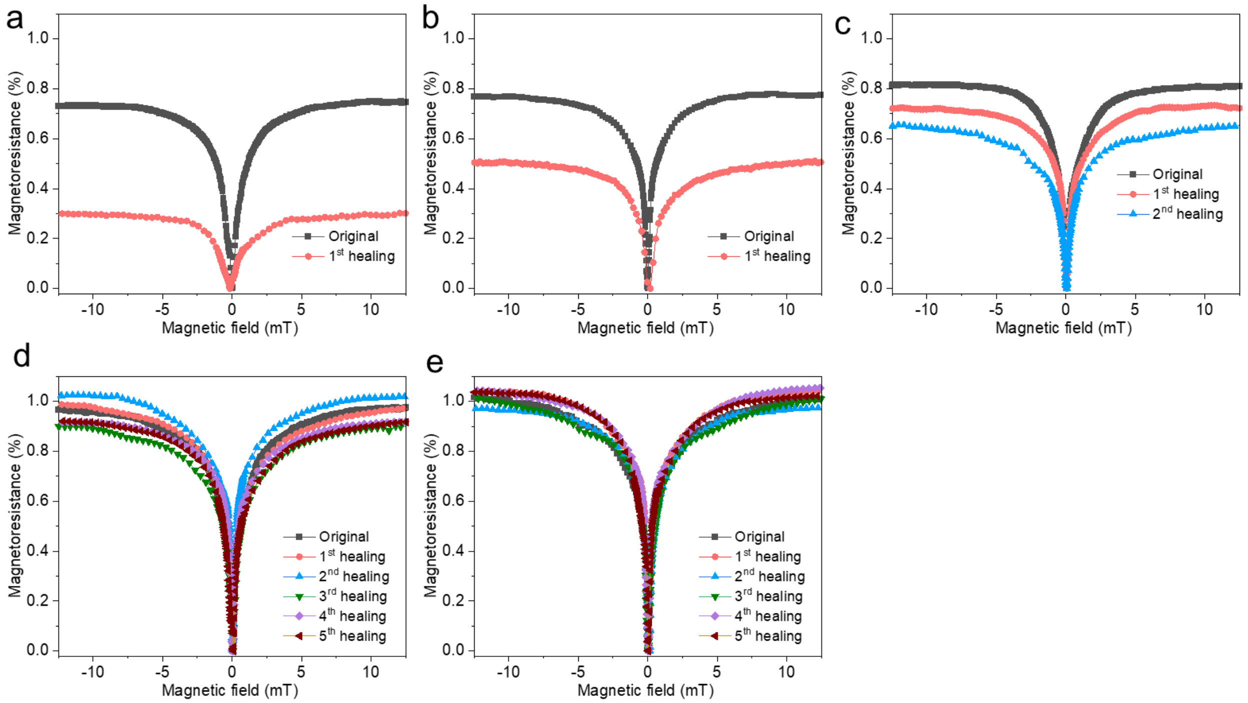

Fig. S9 Self-healing of printed magnetoresistive sensors based on composites of different concentrations of $Ni_{81}Fe_{19}$ microparticles. The concentrations of $Ni_{81}Fe_{19}$ microparticles in the printing solutions (and the corresponding volume fractions in the cured composites) are a) 0.125 g/ml (6.7%), b) 0.15 g/ml (7.7%), c) 0.25 g/ml (12.1%), d) 0.5 g/ml (22.3%), e) 1g/ml (36.5%).

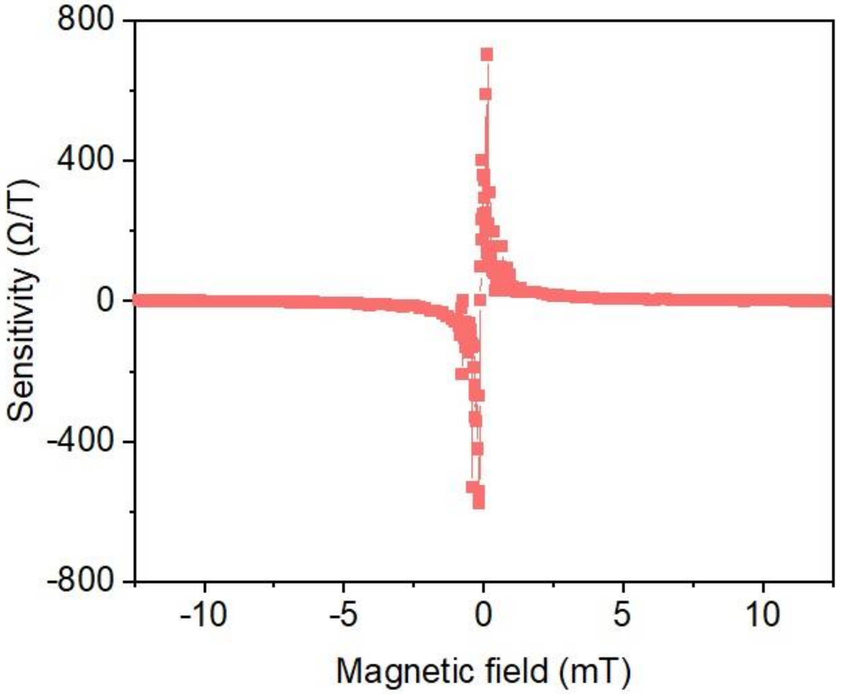

Fig. S10 Sensitivity (*i.e.*, first derivative of the electrical resistance with respect to magnetic field) of printed magnetoresistive element. The measurement resolution of printable magnetoresistive sensor can be defined by the following equation:

$$\text{Resolution} = \frac{\text{noise} \times (\text{corner frequency})^{0.5}}{\text{sensitivity}} = \frac{19\times10^{-6}\Omega\ \text{Hz}^{-0.5}\times(1.80\ \text{Hz})^{0.5}}{700\Omega\ \text{T}^{-1}} \approx 36\ \text{nT}$$

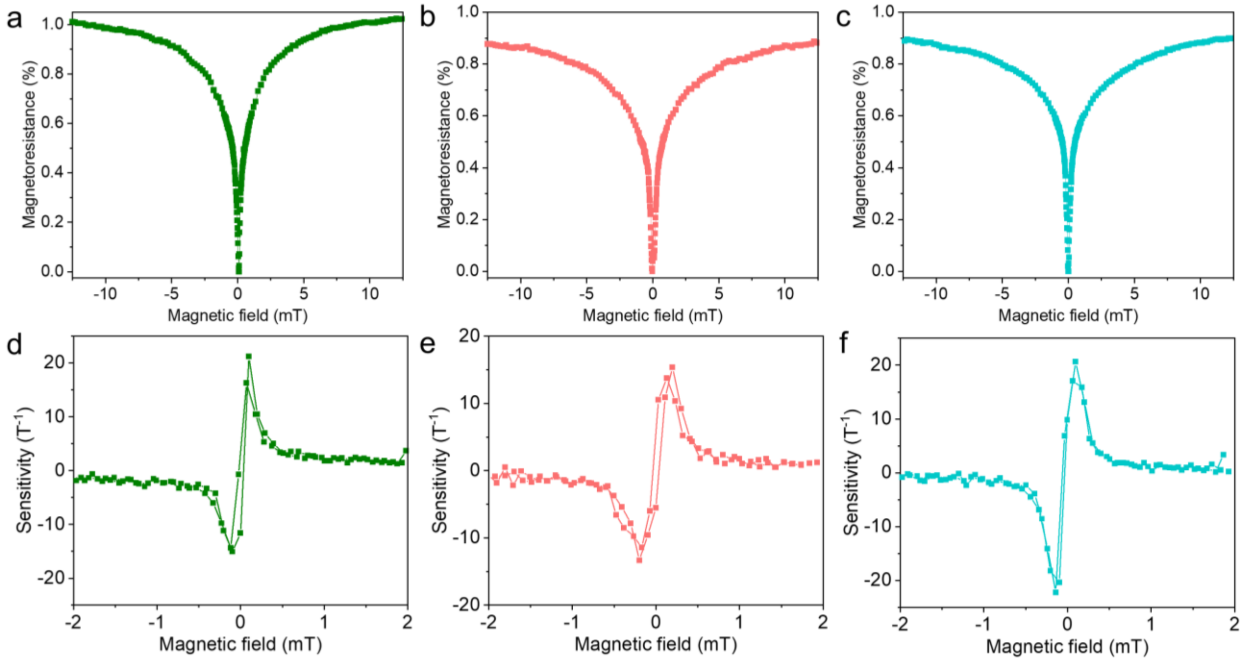

Fig. S11 Characterization of printable magnetoresistive sensors fabricated using different printing techniques. a–c) Magnetoresistance and d-f) magnetoresistive sensitivity of the sensors fabricated using a,d) pipetting, b,e) screen printing, and c,f) spin coating. The original resistances of magnetoresistive element at 0 mT are 11, 14, and 8.2 Ω, respectively.

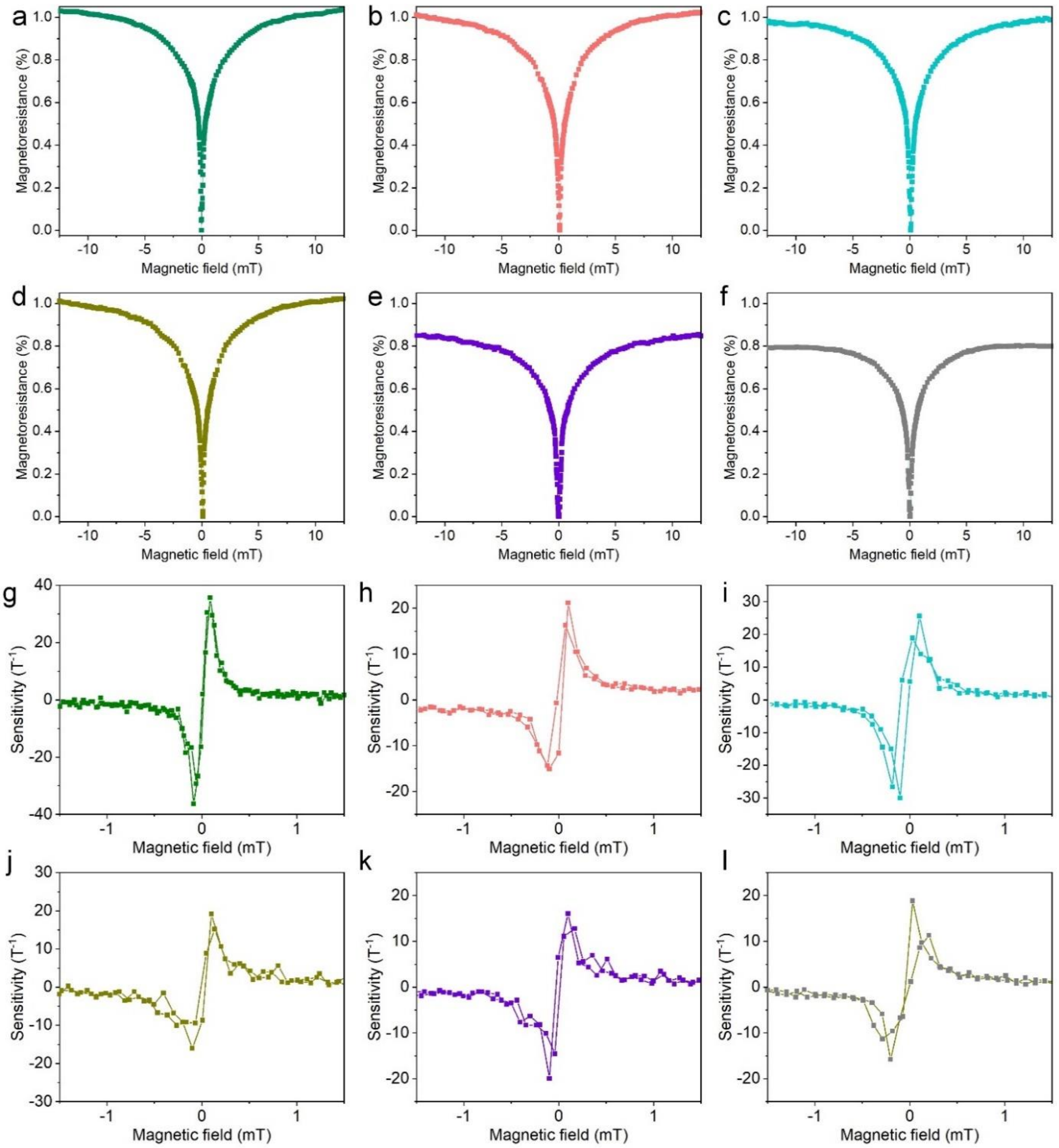

Fig. S12 Characterization of printable magnetoresistive sensors fabricated on different substrates. a–f) Magnetoresistance and g-l) magnetoresistive sensitivity of the sensors fabricated on a,g) flexible flat cables, b,h) Si wafer, c,i) glass, d,j) plastic, e,k) paper, and f,l) ceramic. The original resistances of magnetoresistive element at 0 mT are 7.3, 11, 11.7, 20.3, 24.8 and 20.4 Ω, respectively. The resistance variation among various substrates may be ascribed to different surface roughness that affected $Ni_{81}Fe_{19}$ microparticle alignment as well as different dimensions of magnetoresistive elements.

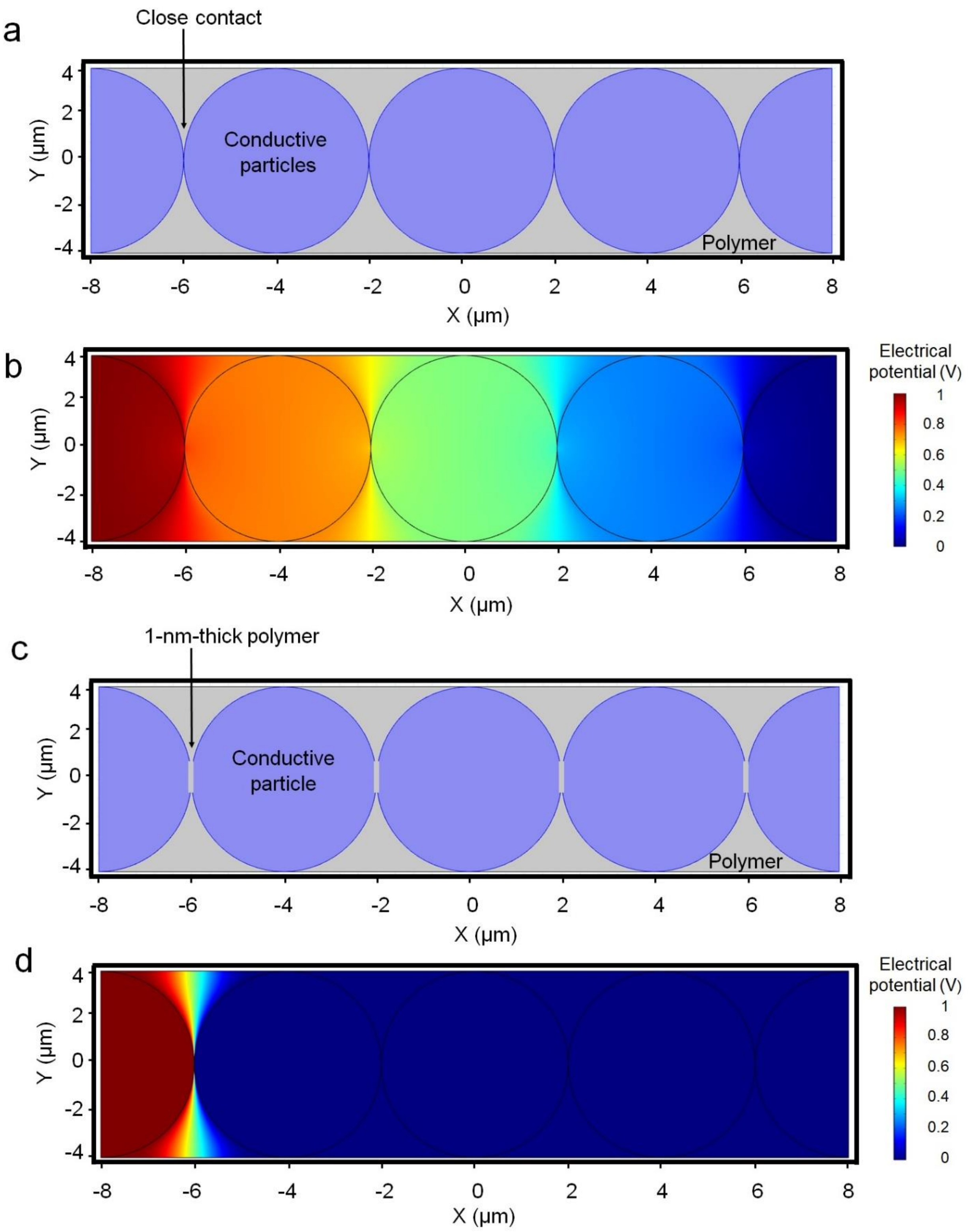

Fig. S13 COMSOL simulation of electric potential. a) Two-dimensional layout of $Ni_{81}Fe_{19}$ microparticles in polymer binder for electrical simulation. For simplification, the microparticle was uniformly set as 4 μm in diameter (identical to that in Fig. 3a,b) according to the size analysis in

Supplementary Fig. 2. Electric potential distribution along the trace of $Ni_{81}Fe_{19}$ microparticles. In b), microparticles are closely in contact with one another. Electric potential decreases evenly along the path. In c), microparticles are isolated by a 1-nm-thick polymer layer and voltage drop localizes in the insulating layer.

Simulation of electric potential in magnetoresistive composite was performed by the ACDC module of COMSOL Multiphysics. The simulation of electrical potential follows stationary current conversion equations:

$$\nabla \cdot J = Q_j \quad (1)$$

$$J = \sigma E + J_e \quad (2)$$

$$E = -\nabla V \quad (3)$$

where J, E, V and σ are the current density, electric field, electric potential and electrical conductivity in the active materials of magnetoresistive composite, respectively; J_e is an externally generated current density.

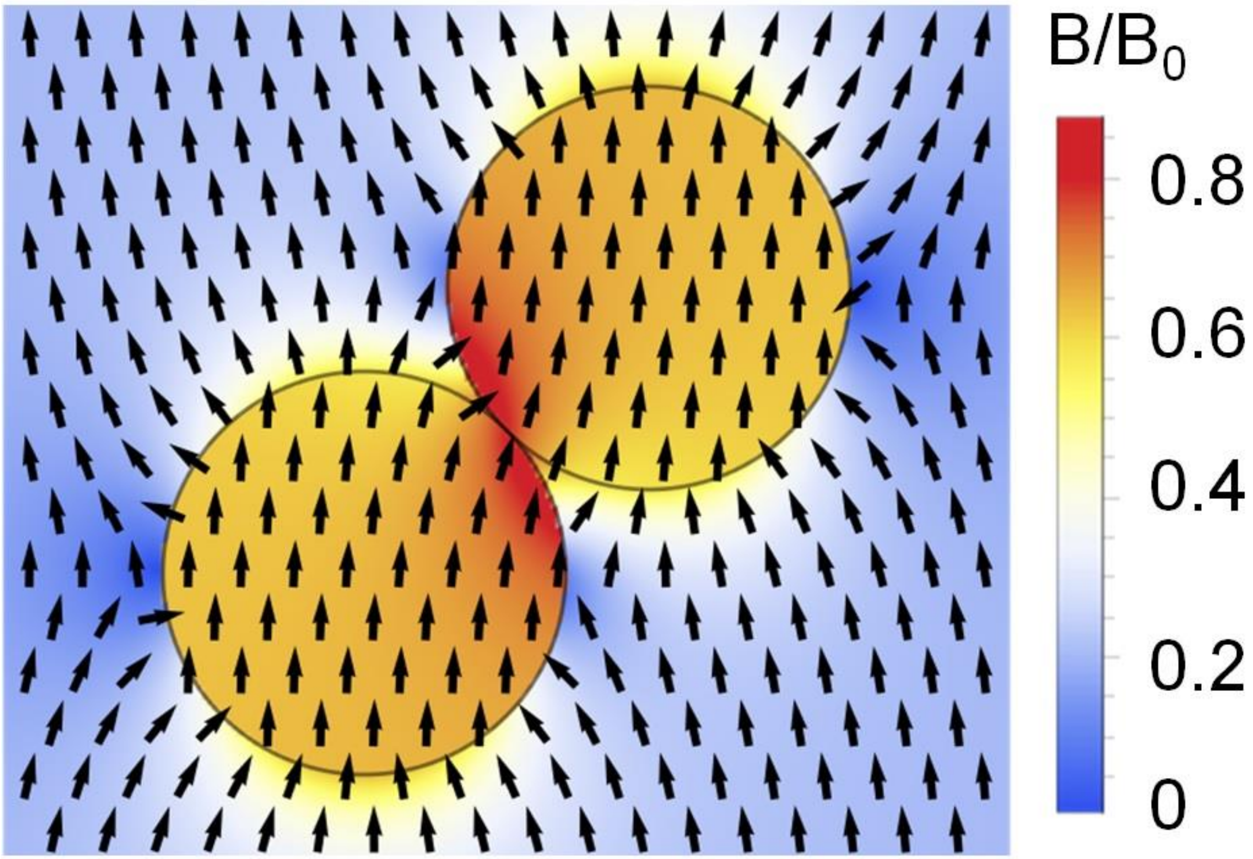

Fig. S14 Simulation for magnetic stray field distribution as two magnetic microparticles are in contact.

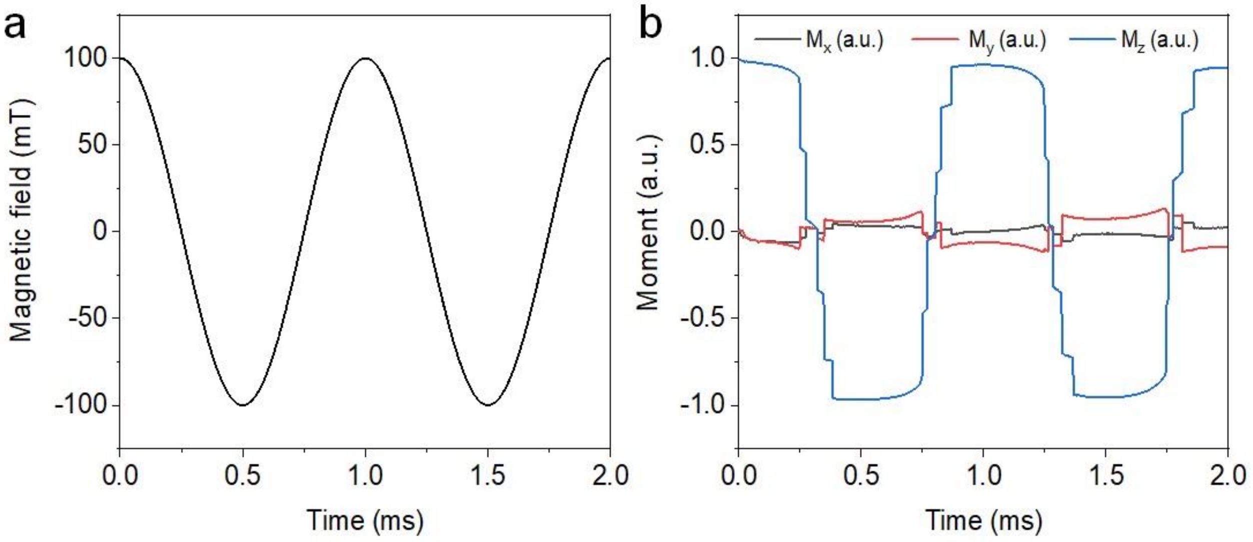

Fig. S15 External magnetic field and magnetic moment as a function time as applying AMF to a system of magnetic particles. Magnetic field is applied along z-axis.

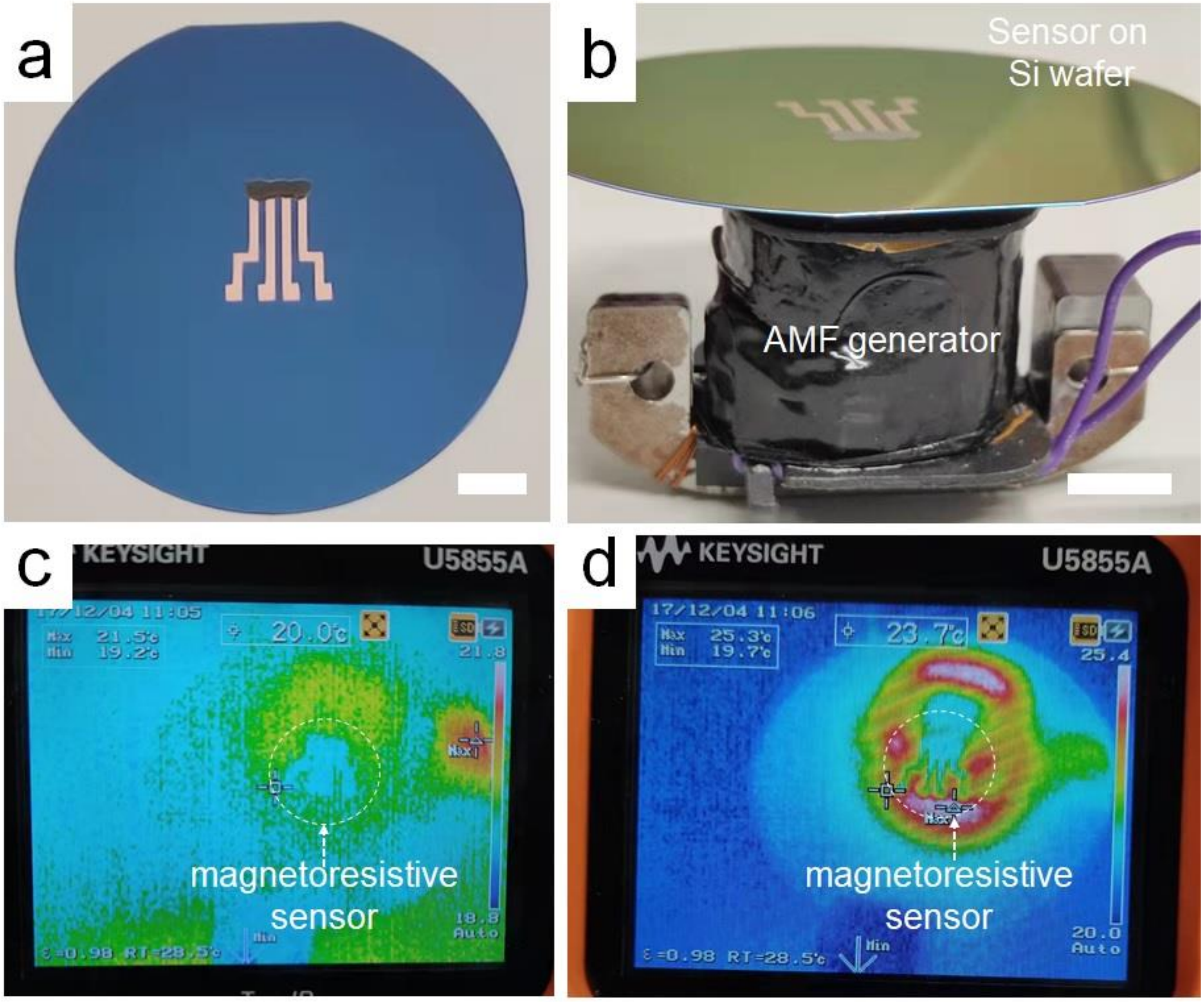

Fig. S16 Temperature characterization of a magnetoresistive sensor in AMF. a) Magnetoresistive sensor fabricated on Si wafer. b) Experimental setup for exposing the magnetoresistive sensor to AFM. Temperature variation c) at room temperature and d) after exposure in AMF for 1 min.

In panels c) and d), the temperature of the sensor was changed from 19.5 to 21.5 ^{o}C after applying AMF for 1 min. The small variation of temperature is in good accordance with magnetic hysteresis loop of magnetoresistive composite in Supplementary Fig. 17, proving that negligible heat was generated in the soft magnetic materials (here, $Ni_{81}Fe_{19}$ microparticles). In other words, the temperature increase (about 2 ^{o}C) mainly stemmed from the heat accumulation in the metal bar of AMF oscillator during operation and thus should not be responsible for the electrical decrease observed in Fig. 1b.

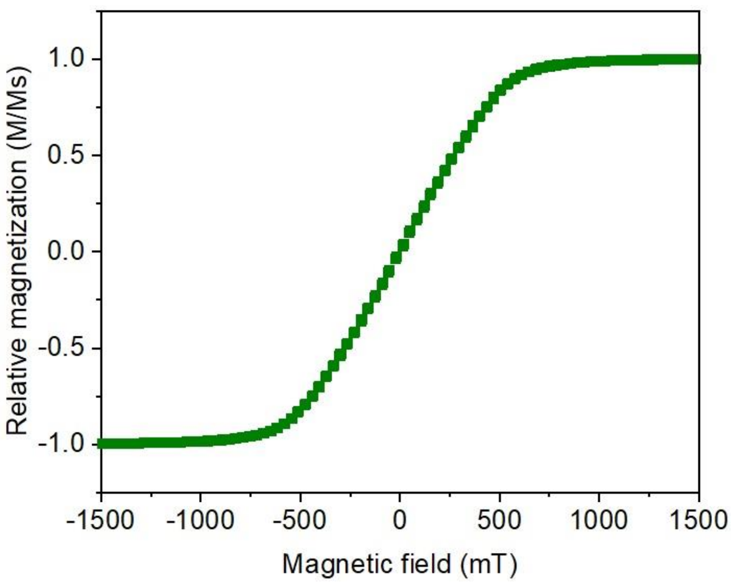

Fig. S17 Magnetic hysteresis loop of the magnetoresistive composite measured in an out-of-plane magnetic field at the temperature of 300 K. Composite was based on solution with 1 g/ml $Ni_{81}Fe_{19}$ microparticles.

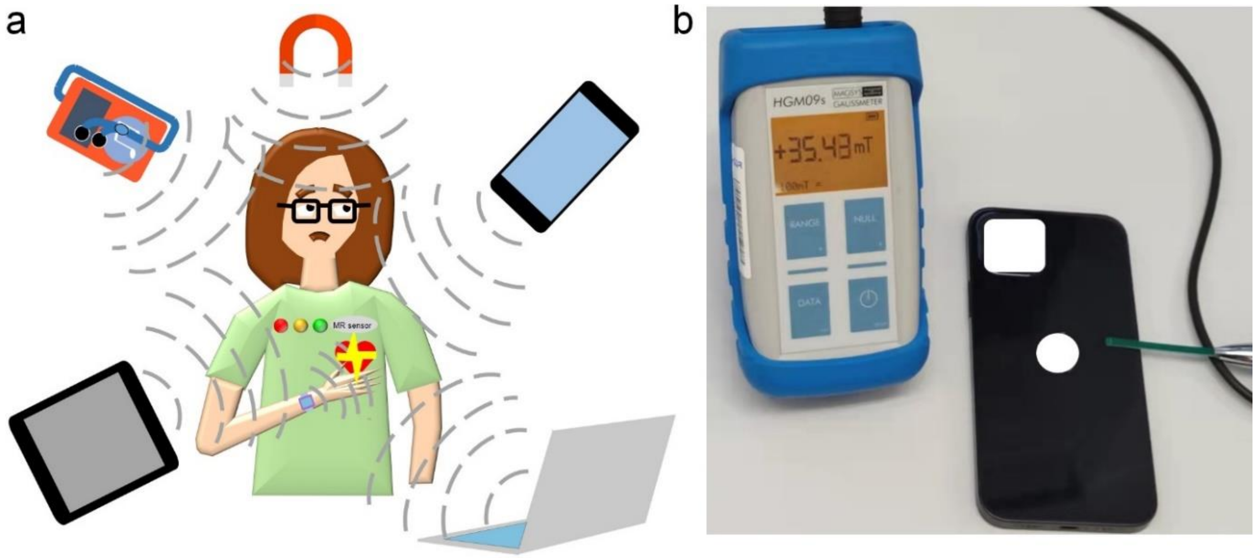

Fig. S18 Magnetic fields experienced by human beings. a) Cartoon for magnetic field generated by electronics in daily life (*e.g.*, MP3, smart phone, smart pad, smart band, and laptop). b) Photograph of a smartphone and magnetic field (of about 35 mT) in the proximity.

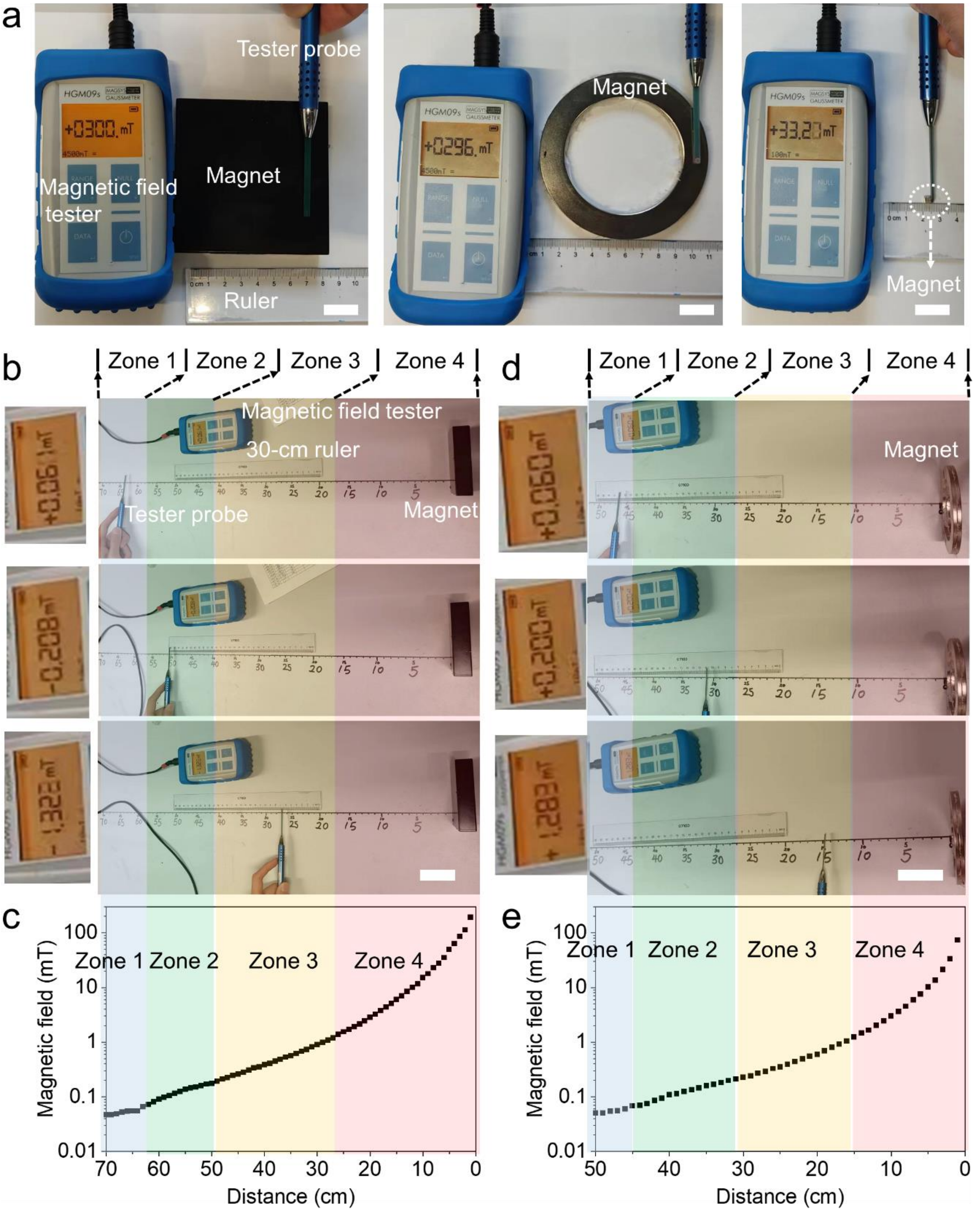

Fig. S19 Magnetic fields around permanent magnets. a) Commercial permanent magnets used for the characterization of the printed magnetoresistive sensors in the safety monitoring. From left to right, a large cube-shaped magnet is of about 10 cm × 10 cm × 2 cm in dimension and 300 mT in magnetic field at its surface; a ring-shaped magnet has 10 cm outer diameter, 8.5 cm inner diameter,

7 mm thickness, and 296 mT magnetic field; a small cube-shaped magnet is of 0.4 cm × 0.4 cm × 0.4 cm in dimension and 33.2 mT in magnetic field. Magnetic field distribution in the proximity of b,c) the large cube-shaped magnet and d,e) the ring-shaped magnet. Based on the magnetoresistance curve of the sensor in Fig. 4d, the magnetoresistance thresholds of 0.1% (corresponding to the boundary of Zone 1 and Zone 2), 0.4% (corresponding to the boundary of Zone 2 and Zone 3), and 0.7% (corresponding to the boundary of Zone 3 and Zone 4) are about 0.06, 0.2, and 1.3 mT. Accordingly, the spatial configuration of Zone 1-4 are marked in c,e). The magnetic field at the corresponding boundaries, measured by a magnetic field tester, is also exhibited in b,d) for a clear understanding of the distance dependence of the magnetic field apart from the permanent magnet. Scale bars are 2 cm in a) and 5 cm in b,d).

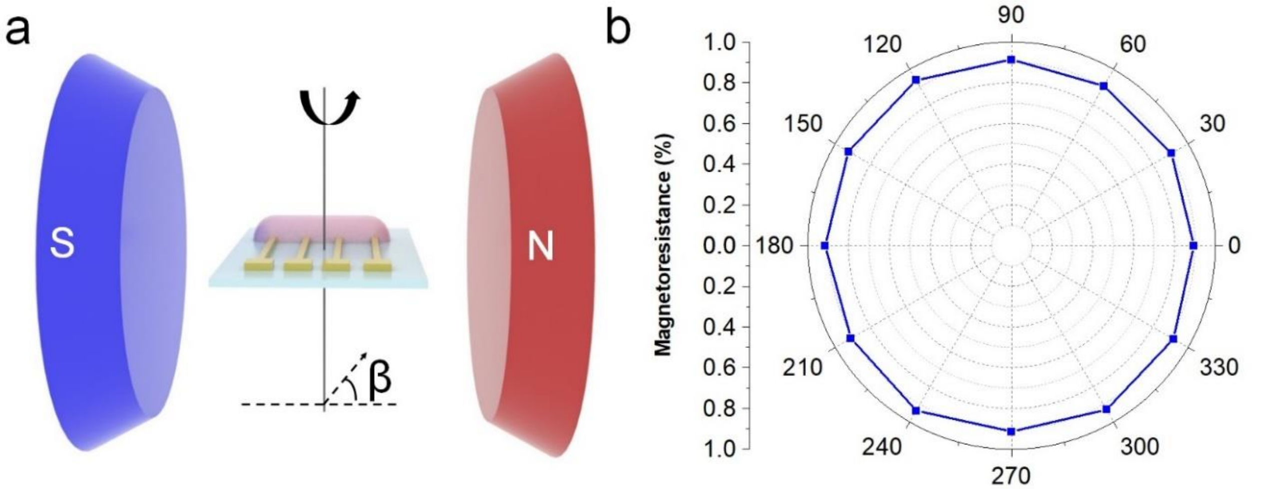

Fig. S20 Angle dependence of magnetoresistance of printed sensors for in-plane measurement. a) Schematic configuration of the experimental setup. b) Magnetoresistance response as changing the magnetic field direction with respect to the magnetoresistance element from 0° to 360°.

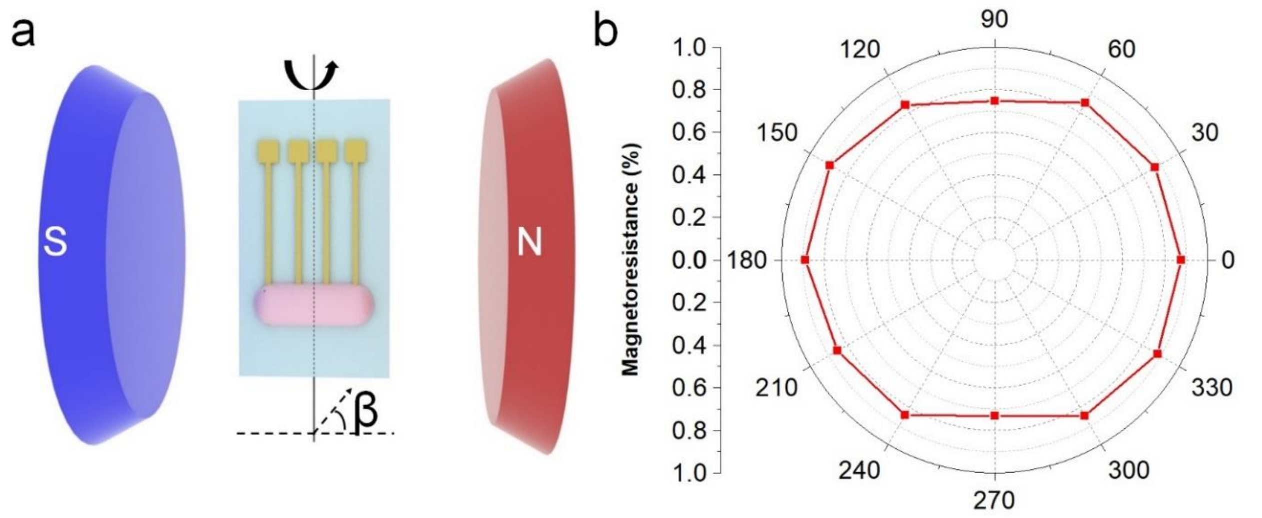

Fig. S21 Angle dependence of magnetoresistance of printed sensors for out-of-plane measurement. a) Schematic configuration of experimental setup. b) Magnetoresistance response as changing the magnetic field direction with respect to the magnetoresistance element from 0° to 360°.

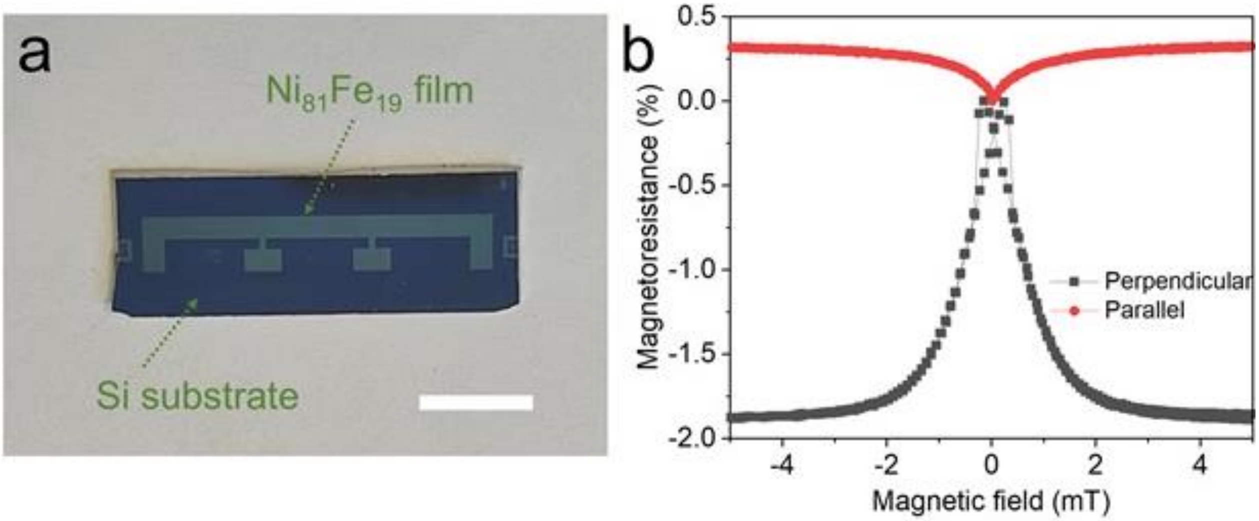

Fig. S22 Characterization of $Ni_{81}Fe_{19}$ thin film sensor. a) Photograph of a $Ni_{81}Fe_{19}$ thin film sensor on Si wafer, scale bar: 5 mm. The $Ni_{81}Fe_{19}$ film of 100-nm thickness was deposited by sputtering at room temperature on a Si wafer with 5-nm Ta as buffer layer. b) Magnetoresistance as a function of magnetic field. The applied magnetic field is perpendicular to or parallel with the electrical path in sensor. Typically, anisotropic magnetoresistance effect of $Ni_{81}Fe_{19}$ thin film sensor is in the range of 1.5-3.0%[1].

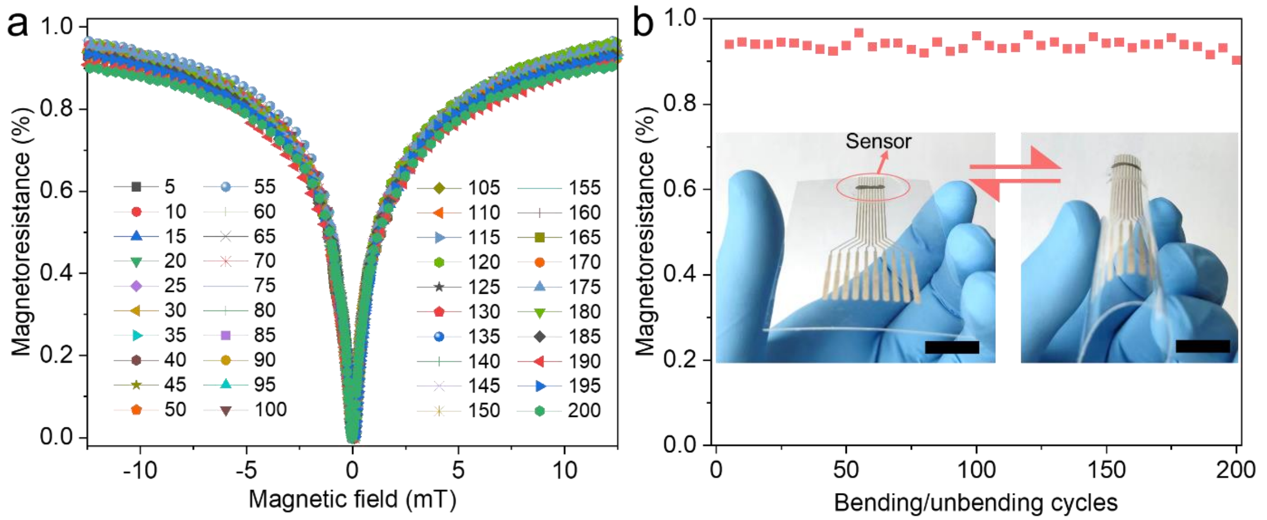

Fig. S23 Operational stability test for the printed magnetoresistive sensor against repeated bending and unbending. a) Magnetoresistance of the printed sensor after repeated bending/unbending cycles. The labels indicate the number of bending/unbending cycles. b) Magnetoresistance of the printed sensor at 12.5 mT as a function of bending/unbending cycles. The magnetoresistances were derived from a). Inset: photographs for the printed sensor in the planar state (right) and the bended state (right). Scale bars: 1 cm.

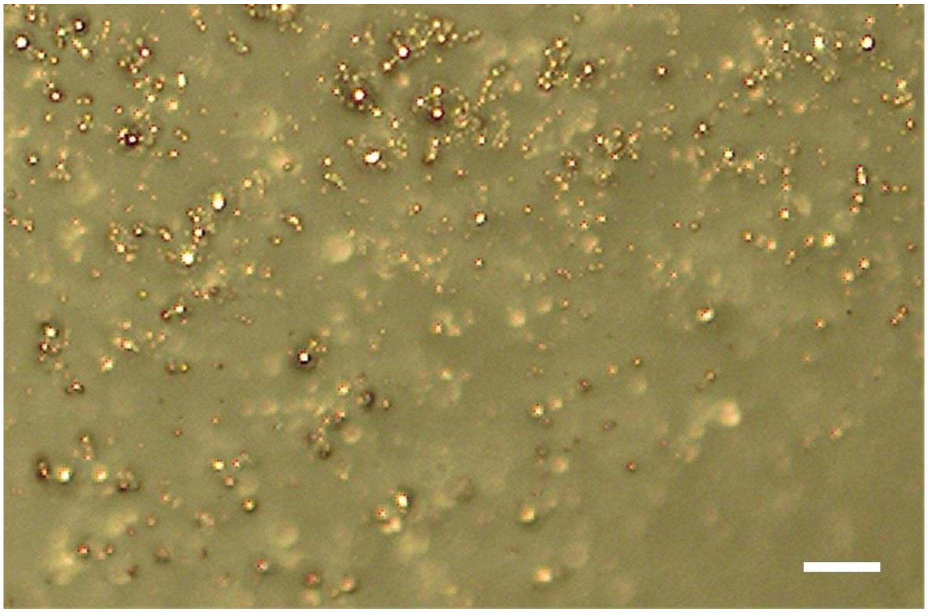

Fig. S24 Top-view optical microscopy image of a cured composite of printed sensor. Scale bar: 50 μm.

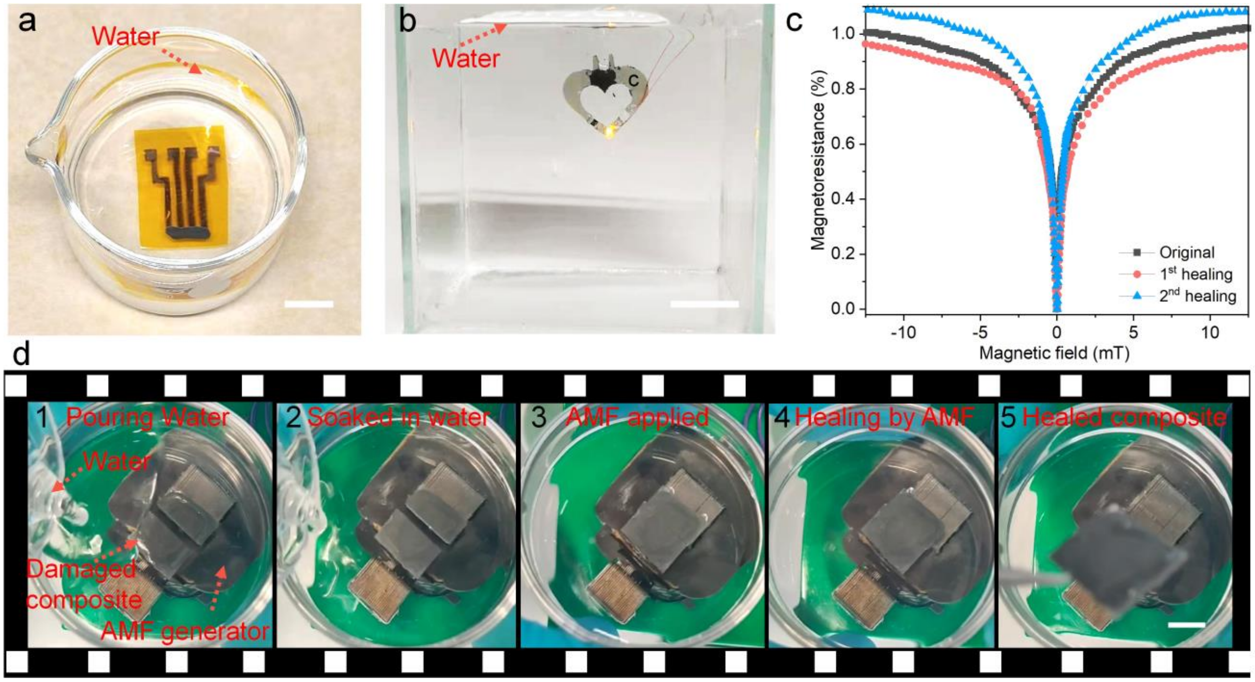

Fig. S25 Magnetoresistive sensors in water. Sensors a) being placed and b) working in water. c) Magnetoresistance variation of a magnetoresistive sensor after carrying out cutting/healing in water for two times. d) Screenshots of Supplementary Movie 3, recording the AMF-mediated self-healing process of a damaged magnetoresistive composite in water (from left to right): d_1) water was poured into a beaker where the damaged composite was placed; d_2) the damaged magnetoresistive composite was completely soaked into water; d_3) two segments of the damaged composite was reconnected, driven by the AMF induced attracting force; d_4) self-healing was carried out through the dynamic reformation of chemical bonds and the entanglement of the polymer chains (driven by AMF-induced $Ni_{81}Fe_{19}$ microparticle oscillations); d_5) the damaged magnetoresistive composite was successfully healed in water. Scale bars: 1 cm.

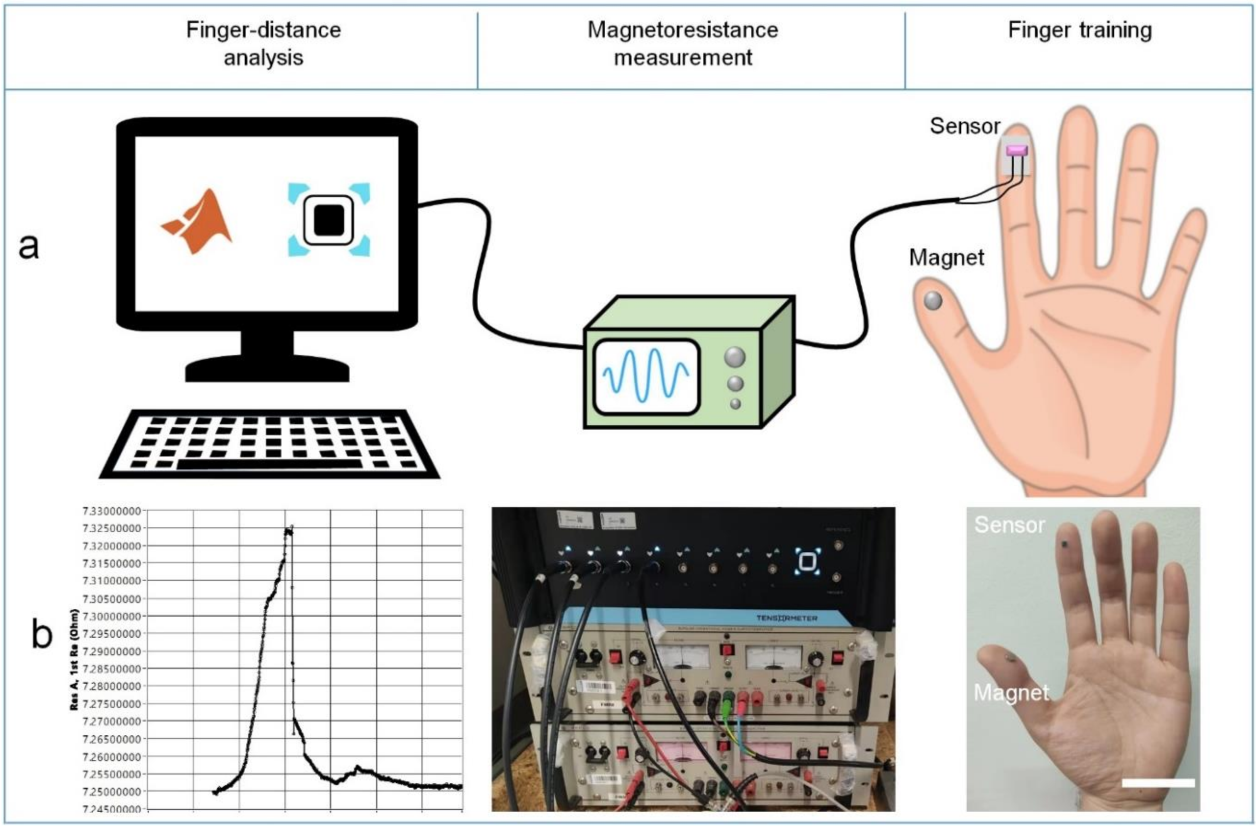

Fig. S26 Implementation of a finger training application using printable magnetoresistive sensor in physical therapy. a) Schematic illustration and b) the corresponding experimental setup and readout. Scale bar: 5 cm.

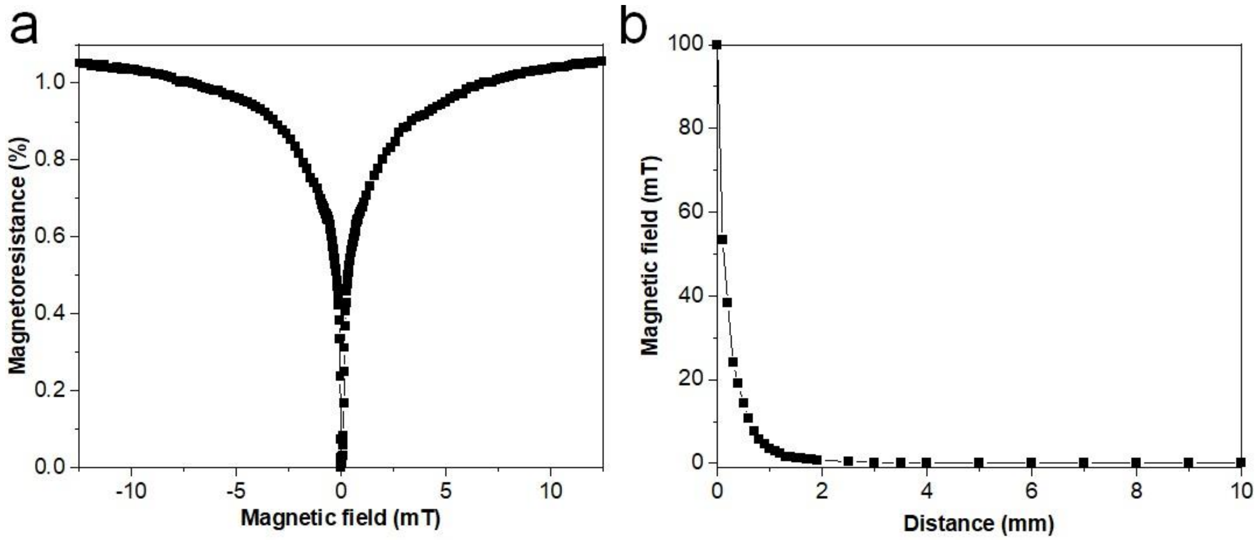

Fig. S27 Characterization of on-skin printable magnetoresistive sensor and permanent magnet used for the finger training application. a) Magnetoresistance of wearable magnetic sensor. b) Magnetic field generated by a permanent magnet as a function of distance.

According to the experimental results in panels a) and b), the relationship between magnetic field (*MF*) and magnetoresistance (*MR*) of printable sensor can be described by equation (1):

$$MR = f\,(MF) \qquad (1)$$

and the magnetic field (*MF*) as a function of distance (*Dist*) generated by a permanent magnet follows equation 2:

$$MF = g\,(Dist) \qquad (2)$$

In other words, as approaching permanent magnet, the printable sensor exhibits a distance dependent magnetoresistance, as defined by equation (3):

$$MR = f\,(g\,(Dist)) \qquad (3)$$

Considering the practical operational situation, the distance and the magnetoresistance in the equation (3) have a one-to-one correspondence. Thus, we have

$$Dist = f^{-1}\, g^{-1}(MR) \qquad (4)$$

Following the equation (4), the magnetoresistance signal can be converted into the distance between two fingers by software of numerical interpolation (here, using a linear interpolation).

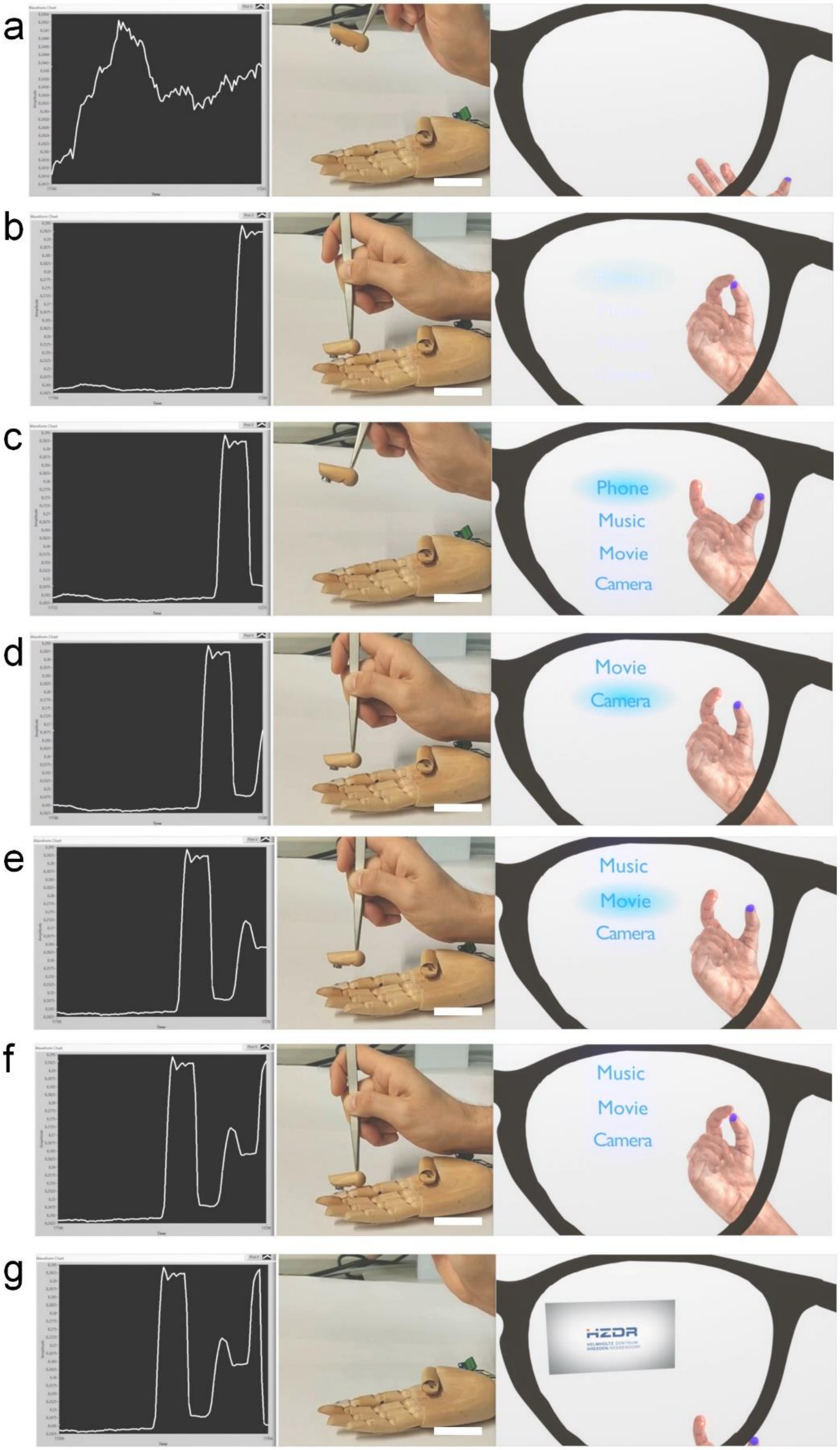
a
b
c
Phone
Music
Movie
Camera
d
Movie
Camera
e
Music
Movie
Camera
f
Music
Movie
Camera
g
HZDR

Fig. S28 Operation of printable magnetoresistive sensor for augment reality (AR). a-g) Snapshots of Supplementary Movie 7: a) as the sensor-mounted forefinger is far away from a magnet mounted on a thumb, the electrical resistance of the magnetoresistive sensor is changed due to surrounding electromagnetic disturbances, which results in the fluctuation noise of the resistance signal. b) As two fingers get in touch, the sensor resistance increases sharply to cross a preset threshold and consequently the system of AR glasses will be started. c) Programs pop up on the lenses. d) As approaching fingers, the sensor resistance is changed. Supposing a one-to-one correspondence between resistance value and programs, various programs can be scanned with changing the distance between forefinger and thumb. e) At a specific distance, the program of interest (here, video player) is selected after holding the fingers for several seconds. f) As two fingers are in contact again and the sensor resistance becomes higher than the threshold value, the program of video player is activated. g) Video is playing on the lenses. Scale bars: 5 cm.

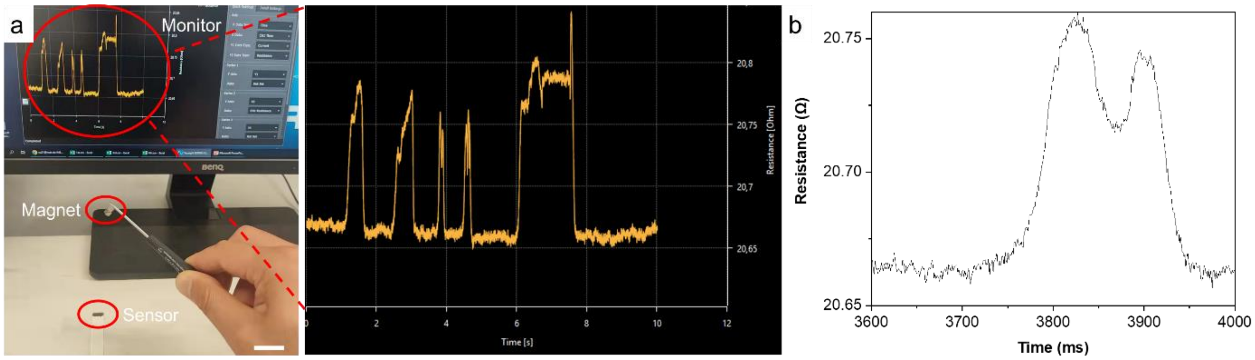

Fig. S29 Demonstration for the response speed of the printed magnetoresistive sensor. a) Experimental setup (including a monitor, a permanent magnet, and the magnetoresistive sensor) and the magnetoresistance responsive curve with the magnet approaching the sensor for five times at different speeds. The third pulse of the magnetoresistance curve in a) are plotted in b) for clear observation. Scale bar: 3 cm.

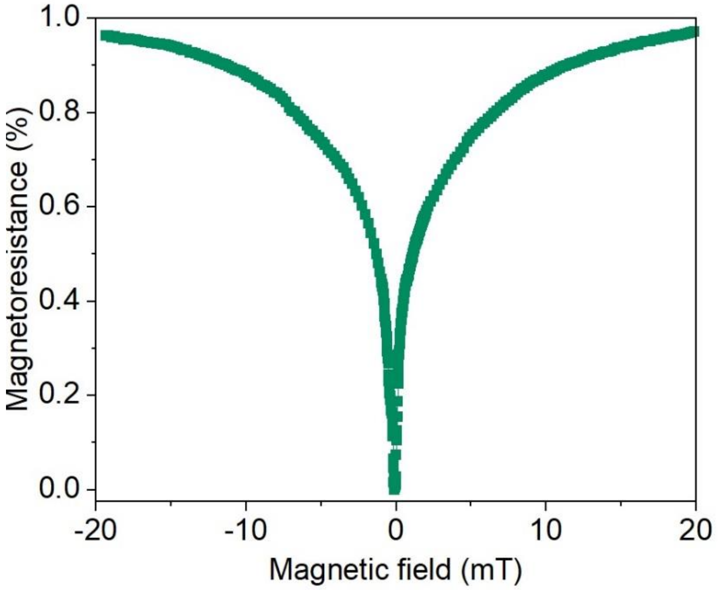

Fig. S30 Characterization of printable magnetoresistive sensor using $Ni_{81}Fe_{19}$ microparticles as fillers and poly(styrenebutadiene-styrene) as polymeric binder. To form binder solutions, 1.5 grams of polymer was dissolved into 10 milliliters of Xylol and magnetically stirred for 10 hours at room temperatures. Other fabrication procedures and magnetic characterization were the same. The electrical resistance of the magnetoresistive element at 0 mT is 7.5 Ω.

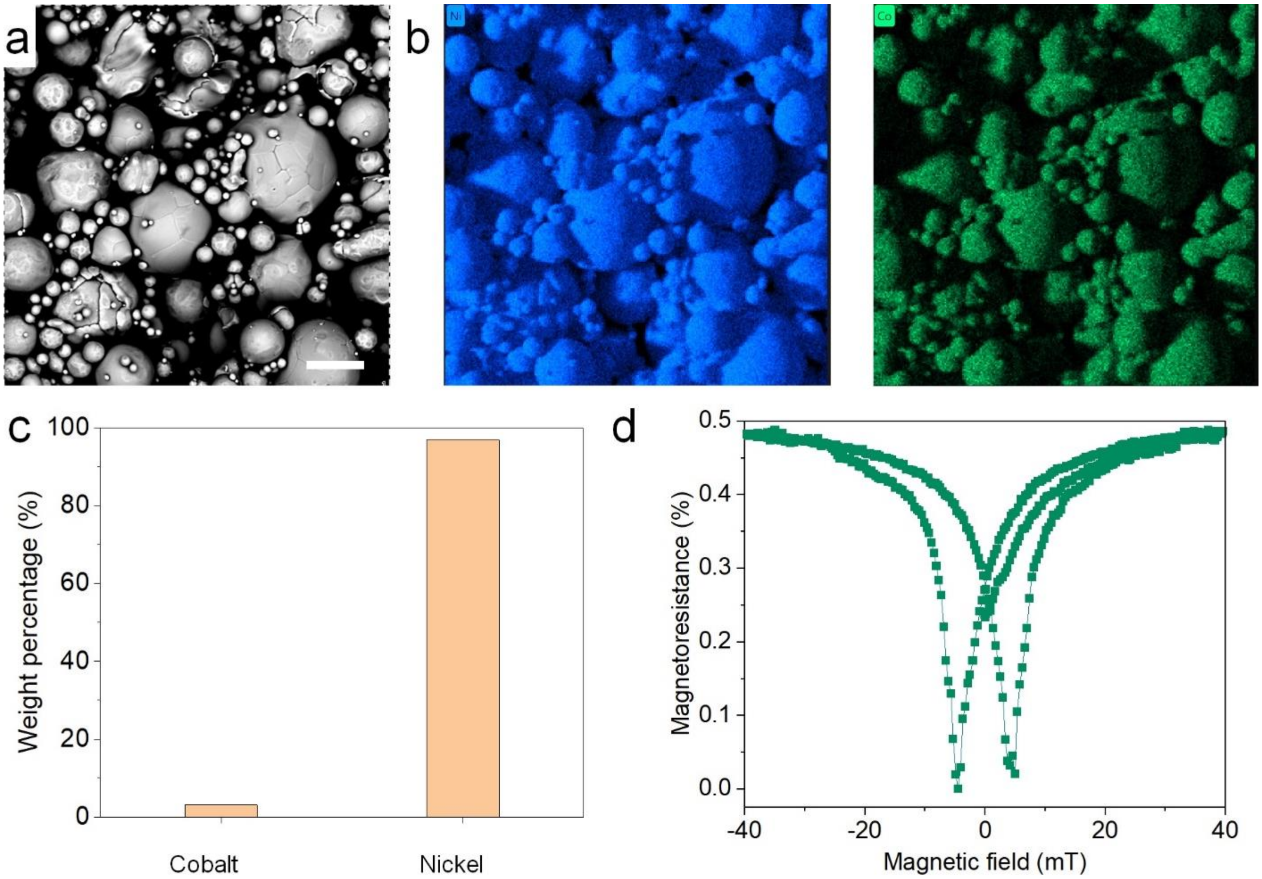

Fig. S31 Characterization of $Ni_{97}Co_3$-composite based magnetoresistive sensor. a) SEM image of $Ni_{97}Co_3$ microparticles. b) EDX maps and c) weight percentage of elements of Ni and Co in $Ni_{97}Co_3$ microparticles. d) Magnetoresistance of $Ni_{97}Co_3$-composite based magnetoresistive sensor. Scale bar: 10 μm.

The fabrication process and magnetic characterization of $Ni_{97}Co_3$-composite based magnetoresistive sensor is the same as that used for printable $Ni_{81}Fe_{19}$ magnetoresistive sensor. An arbitrary 1 g/ml was used for the filler concentration of composite solution. The electrical resistance of the magnetoresistive element at 0 mT is 24.8 Ω.

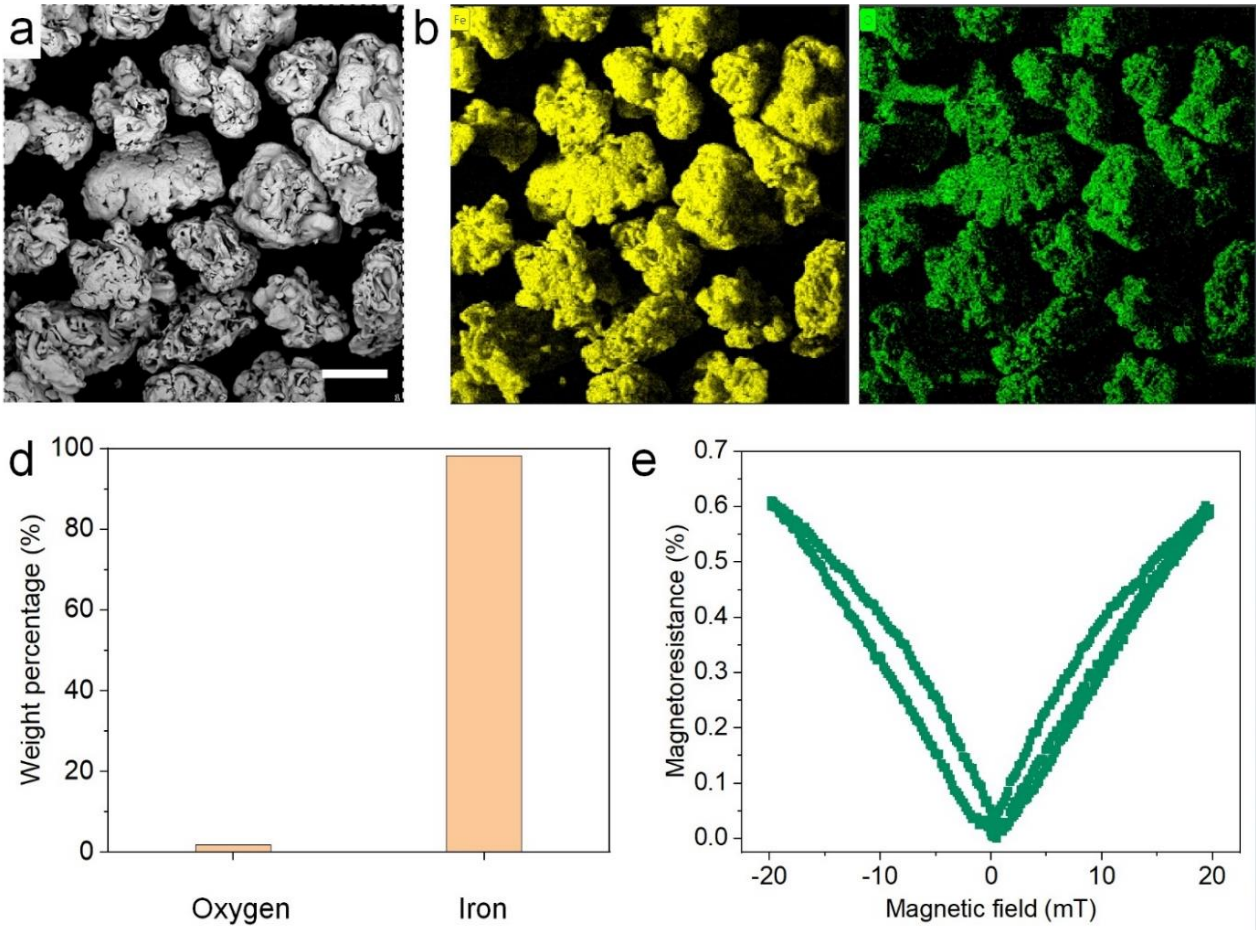

Fig. S32 Characterization of Fe-composite based magnetoresistive sensor. a) SEM image of Fe microparticles. b) EDX maps and c) weight percentage of elements of Fe and O in Fe microparticles. d) Magnetoresistance of Fe-composite based magnetoresistive sensor.
Scale bar: 100 μm.

The fabrication process and magnetic characterization of Fe-composite based magnetoresistive sensor is the same as that used for printable $Ni_{81}Fe_{19}$ magnetoresistive sensor. An arbitrary 1 g/ml was used for the filler concentration of composite solution. The presence of oxygen element in EDX map (panel c)) came from the oxidization of iron at the microparticle surfaces. Although the thin oxide layer was adverse to the electrical percolation between neighboring Fe microparticle, the AMF-induced intimate connection still can overcome this problem and generate magnetoresistive performance. The electrical resistance of the magnetoresistive element at 0 mT is 12.4 Ω.

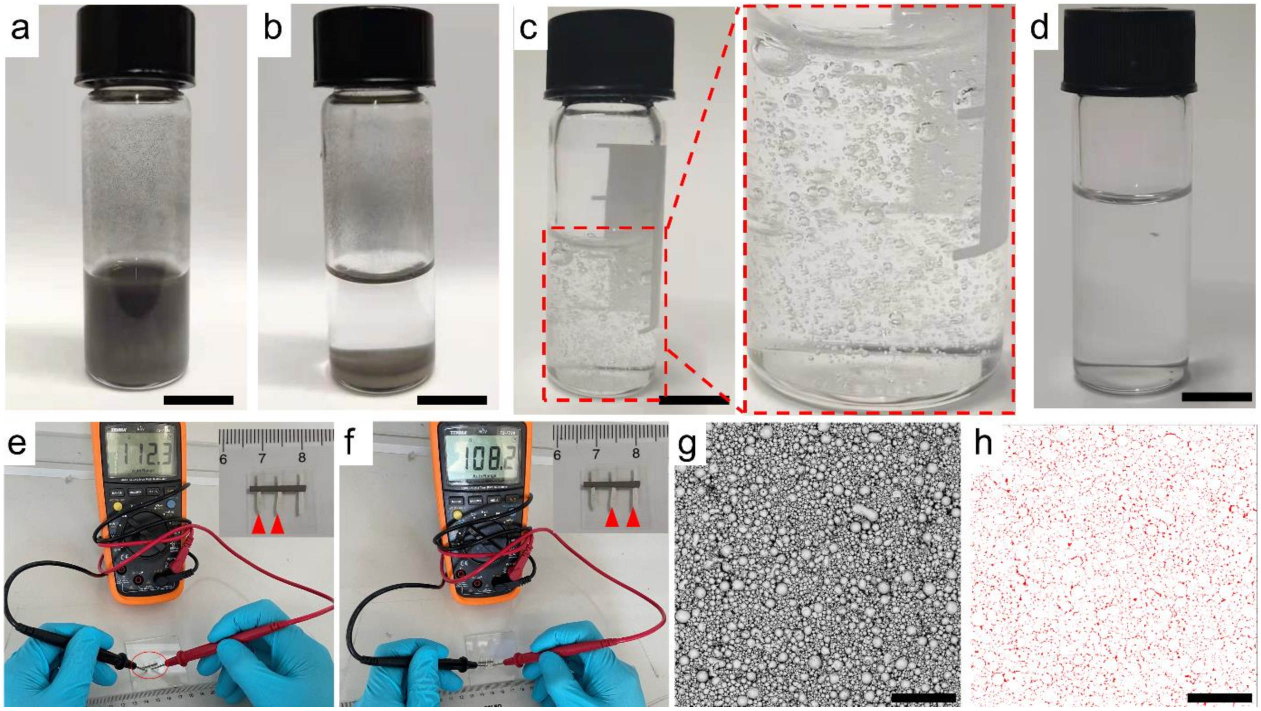

Fig. S33 Magnetoresistive composite. Composites a) with and b) without solvents after shaking. Polymeric binders c) with and d) without solvents after agitating. For a clear observation of the generated air bubbles, $Ni_{81}Fe_{19}$ microparticles are not mixed into the polymer binder. e,f) Electrical resistances for two segments of a printed composite trace. Insets: photography of the printed composite. g) SEM images of the printed composite after plasma etching. h) Voids without microparticles (denoted by red dots) derived from g). Statistically, the surface coverage of void is about 5.6%. Scale bars are 1 cm in a) – d) and 200 μm in g) – h).

As the polymeric binders of PDMS and PBS were dissolved in a solvent, the viscosity of the binder solutions can be tuned that is desirable for dispersion of $Ni_{81}Fe_{19}$ microparticles in the whole volume after shaking (Supplementary Fig. 33a). In contrast, as the binders were not diluted by the solvent, the composite exhibited very high viscosity. Consequently, $Ni_{81}Fe_{19}$ microparticles cannot be dispersed by shaking and always concentrated at the bottom of the composite (Supplementary Fig. 33b). It is worth noting that because of the high viscosity, air bubbles were easily generated in the binder without solvent during agitating that are not visually observed in the low-viscosity binder solutions diluted by solvent (Supplementary Fig. 33c,d). Therefore, the addition of solvent into the composite is crucial for forming the electrical percolation pathways in the following printing steps. Supplementary Fig. 33e, f compare the electrical resistances of two segments of a printed trace based on the diluted binders. Two resistance values for the same length of 5 mm are 112.3 and 108.2 Ω, respectively. Considering the roughness variation of the plastic substrate, the small resistance deviation of about 2% indicates the homogeneous dispersion of microparticles in the printed composite. The homogeneity of the microparticle dispersion can be further verified by SEM images of the printed composite in which no obvious agglomeration of microparticles is observed (Supplementary Fig. 33g). Statistical analysis points out that voids without $Ni_{81}Fe_{19}$ microparticles

only account for a 5.6% surface coverage. In particular, these voids are randomly distributed in the whole space and isolated from each other, thus avoiding the blocking of electrical conductance along the printed trace.

Supplementary references

1 Heremans, J. Solid state magnetic field sensors and applications. *J. Phys. D: Appl. Phys.* **26**, 1149 (1993).

2 Tang, M. et al. Autonomous self-healing, self-adhesive, highly conductive composites based on a silver-filled polyborosiloxane/polydimethylsiloxane double-network elastomer. *J. Mater. Chem. A,* **7**, 27278-27288 (2019).

3 D'Elia, E. et al. Self-healing graphene-based composites with sensing capabilities. *Adv. Mater*. **27**, 4788–4794 (2015).
