# Supplementary material for: Self-healable printed magnetic field sensors using alternating magnetic fields
Source: Nat Commun. 2022 Nov 3;13:6587. doi: 10.1038/s41467-022-34235-3 (PMC9631606; doi:10.1038/s41467-022-34235-3)
Supplement: Supplementary file 2 — Description of Additional Supplementary Files [file 41467_2022_34235_MOESM2_ESM.pdf]

Title: Supplementary Dataset 1:
Description: This file contains data to plot graphs in figure1.

Title: Supplementary Dataset 2:
Description: This file contains data to plot graphs in figure 2.

Title: Supplementary Dataset 3:
Description: This file contains data to plot graphs in figure 4.

Title: Supplementary Dataset 4:
Description: This file contains data to plot graphs in figure 5.

Title: Supplementary Movie 1.
Description: AMF assisted self-healing of magnetoresistive paste with a micrometer-scaled crack.

Title: Supplementary Movie 2.
Description: AMF assisted self-healing of magnetoresistive sensor connected into a circuit.

Title: Supplementary Movie 3.
Description: AMF assisted self-healing of magnetoresistive sensor with a millimeter-scaled gap submerged into deionized water.

Title: Supplementary Movie 4.
Description: Dynamics of 27 magnetic particles in 1 kHz magnetic field.

Title: Supplementary Movie 5.
Description: Interaction of magnetic microparticles in paste under AMF.

Title: Supplementary Movie 6.
Description: Printed magnetoresistive sensor for safety application.

Title: Supplementary Movie 7.
Description: Printed magnetoresistive sensor for human- machine interface in augmented reality.

Title: Supplementary Movie 8.
Description: Different responsive speeds.
